# Spatiotemporal patterns of sortilin and SorCS2 localization during organ development

**DOI:** 10.1186/s12860-016-0085-9

**Published:** 2016-03-10

**Authors:** Simon Boggild, Simon Molgaard, Simon Glerup, Jens Randel Nyengaard

**Affiliations:** MIND Centre, Stereology and Electron Microscopy Laboratory, Aarhus University, 8000 C Aarhus, Denmark; MIND Centre, Department of Biomedicine, Aarhus University, Ole Worms Allé 3, 8000 C Aarhus, Denmark; Centre for Stochastic Geometry and Advanced Bioimaging, Aarhus University, 8000 C Aarhus, Denmark

**Keywords:** Sortilin, SorCS2, Development, Localization, Expression, Vps10p

## Abstract

**Background:**

Sortilin and SorCS2 are part of the Vps10p receptor family. They have both been studied in nervous tissue with several important functions revealed, while their expression and possible functions in developing peripheral tissue remain poorly understood. Here we deliver a thorough characterization of the prenatal localization of sortilin and SorCS2 in mouse peripheral tissue.

**Results:**

Sortilin is highly expressed in epithelial tissues of the developing lung, nasal cavity, kidney, pancreas, salivary gland and developing intrahepatic bile ducts. Furthermore tissues such as the thyroid gland, developing cartilage and ossifying bone also show high expression of sortilin together with cell types such as megakaryocytes in the liver. SorCS2 is primarily expressed in mesodermally derived tissues such as striated muscle, adipose tissue, ossifying bone and general connective tissue throughout the body, as well as in lung epithelia. Furthermore, the adrenal gland and liver show high expression of SorCS2 in embryos 13.5 days old.

**Conclusions:**

The possible functions relating to the expression patterns of Sortilin and SorCS2 in development are numerous and hopefully this paper will help to generate new hypotheses to further our understanding of the Vps10p receptor family.

## Background

Sortilin and SorCS2 are members of the Vps10p-domain-containing receptor family [1, 2]. Sortilin is a multi-ligand receptor shown to function at the level of the Trans Golgi Network (TGN), plasma membrane and lysosomes in sorting a variety of molecules [3]. Functions of sortilin have been investigated in the central and peripheral nervous system (CNS and PNS) and include trafficking and signaling of neurotrophins (NTs) and their receptors [4, 5]. In particular, the interaction between sortilin, the pro-versions of nerve growth factor (pro-NGF), brain derived neurotrophic factor (pro-BDNF), NT-3, the NT receptor p75^NTR^ and the resulting apoptotic signals have been well examined [6–10]. SorCS2 has been studied considerably less but focus has also been on nervous structures. A recently published paper from our group has established that SorCS2 exists in CNS and PNS in single- and two-chain forms that regulate dopaminergic axon guidance and peripheral sensory neuron apoptosis, respectively, and interact with pro-BDNF/p75^NTR^ [2]. Interestingly, SorCS2 has also been shown to be essential for pro-NGF mediated growth cone collapse [11]. Variations in the sortilin gene have been implicated in the processing of amyloid precursor protein (APP) and essential tremor and variations in the SorCS2 gene have been shown to occur more frequently in bipolar disorder, schizophrenia and ADHD suggesting both sortilin and SorCS2 as important for normal CNS function [12–17]. In addition, recent findings have indicated that sortilin and SorCS2 may be associated with increased risk of Alzheimer’s disease [18]. Research into the physiological role of sortilin outside the nervous system has found sortilin to be involved in trafficking of glucose transporter type 4 vesicles [19] and expressed in several types of cancer cells [20–22]. Furthermore, GWAS studies have found an allele of the sortilin gene locus that increases hepatic sortilin expression and confers a protective effect against cardiovascular disease [23, 24]. Subsequent studies of this have produced results suggesting that increased expression of hepatic sortilin reduces levels of low density lipoprotein (LDL)-cholesterol in blood, and thereby atherosclerosis [25]. Surprisingly, lack of sortilin also leads to decreased LDL-cholesterol levels in blood through decreased VLDL secretion [26]. To complicate matters further, sortilin has been identified as a facilitator of proprotein convertase subtilisin/kexin type 9 (PCSK9) secretion, a protein that reduces LDL-receptor expression [19, 27]. Consequently, the exact role of sortilin in cholesterol metabolism remains a matter of debate. Sortilin and SorCS2 are expressed dissimilarly in the CNS, but seem to have partly overlapping functions, with both being able to transmit apoptotic signals via pro-BDNF and pro-NGF [2, 7]. This relationship of structural and in part functional similarities combined with complementary expression patterns might be found outside the CNS as well. Furthermore, the functions of SorCS2 and sortilin with these neurotrophins are relevant for neurodevelopment and conceivably so for the maturation of other cell systems. Thus, it is relevant to study sortilin and SorCS2 together in non-neuronal tissues. While there is data concerning the expression of sortilin and SorCS2 mRNA during development, the focus has so far been on the nervous system. Here, we deliver a thorough description of the spatiotemporal localization of sortilin and SorCS2 in organs and peripheral tissue of the developing mouse embryo, hopefully enabling new hypotheses toward their physiological roles to be created and tested.

## Methods

### Animals

Animals used for this study were C57j/bl6bom mice. Sortilin(−/−) and SorCS2(−/−) mice were bred on C57j/bl6bom background using homologous recombination [4]. All animals were bred and housed at the Animal Facility at Aarhus University in accordance with the Danish Animal Protection Act (12/09/2015). A maximum of five mice per plastic cage (42 x 25 x 15 cm) were housed with water ad libitum and fed standard chow (Altromin #1324). Cages contained nesting material, bedding, a metal tunnel and a wooden stick and were cleaned every week. Animals were kept under pathogen-free conditions with a 12-h light/12-h dark schedule. Experiments were approved by the Danish Animal Experiments Inspectorate (Permit 2011/561–119). For the expression analysis three embryos from two different mothers were used for each age point.

### Tissue

Fetal tissue was obtained through timed-pregnant C57j/bl6bom mice, on embryonic day (E) 13.5, 15.5 and 17.5. Midday on the day of vaginal plug detection was determined as E0.5. Pregnant mice were euthanized by cervical dislocation, the uterus removed and transferred to ice cold phosphate buffered saline (PBS). Embryos were dissected out and fixed in 4 % paraformaldehyde for 1–2 days. After cryoprotection in 30 % sucrose, embryos were covered in Tissue-Tek (Sakura, 4583) and immersed in liquid nitrogen. Sagittal and horizontal sections were cut at 10 μm using a cryostat (Microm HM 500 OM) and stored at −20 °C until use. Adult SorCS2(−/−) and sortilin(−/−) mice have no gross abnormalities of their organs. Therefore a presumption that SorCS2 and sortilin do not play a crucial role in the early organogenesis was made. Age points E13.5, E15.5 and E17.5 were thus selected as the different organs here are relatively easy to identify and important maturational processes take place.

### Immunohistochemistry

Antigen-retrieval was performed by microwaving the tissue for 2 × 5 min at 540 W in Target Retrieval Solution (DAKO, s1699) using a 1:10 dilution in PBS. Sections were cooled to room temperature, followed by 3x10 min wash in PBS. To permeabilize the cells and block endogenous reactivity, sections were incubated with PBS containing 1 % bovine serum albumin (BSA; Sigma, A4503) and 0.3 % Triton X-100 (Sigma, T8787) for 30 min. After a 10 min washing step, primary antibodies were added in a 50 nM Tris-based (TB; 6.06 g Tris, 8.77 g NaCl in 1 L H_2_O, pH 7.4) buffer solution containing 1 % BSA and sections placed overnight at 4 °C. The next day, sections were left at room temperature for 1 h followed by 3 × 10 min of wash in PBS. Secondary antibodies were added in TB and sections left in the dark for 4 h at room temperature before washing 3 × 10 min. Nuclei were visualized by incubating with Hoechst stain using a 1:10,000 dilution in PBS for 10 min. Sections were mounted using fluorescent mounting medium (DAKO, s3023) and kept in the dark at 4 °C. Primary antibodies used were goat α-sortilin (R&D systems, AF2934,1:100), sheep α-SorCS2 (R&D systems, AF4237, 1:50), rabbit α-TTF-1 (Santa Cruz, H-190,1:250), rabbit α-calretinin (Milipore, ab5054, 1:250), rabbit α-Calbindin (Milipore, ab1778, 1:50) and rabbit α-Tyrosine Hydroxylase (Milipore, AB152, 1:250). Reviews of all antibodies used have been made public on the antibody rating site http://www.pabmabs.com. These extra primary antibodies were selected during the study to elaborate on observed sortilin and SorCS2 expression. Secondary antibodies used were donkey α-sheep 488, donkey α-goat 488 and donkey α-rabbit 565 (Life Technologies, A-11015, A-11055, A-10042, 1:300).

Images were captured using a Zeiss LSM780 laser-scanning confocal microscope controlled by ZEN 2011 software. Specificity of sortilin and SorCS2 antibodies were ensured using sortilin(−/−) and SorCS2(−/−) tissue [2, 5]. The expression level and localization of sortilin and SorCS2 was determined visually.

## Results

### Sortilin

#### Respiratory tract

##### Nose/upper airways

**Fig. 1:**
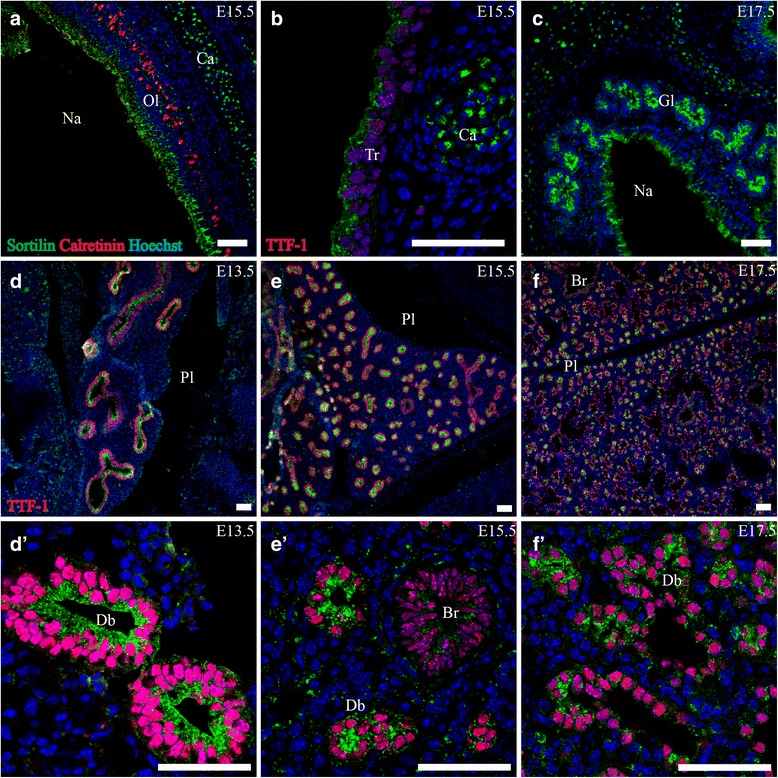
Sortilin expression in embryonic respiratory tract. Immunofluorescence was performed on sagittal sections (10 μm) of embryos on embryonic day E13.5 (**d**), E15.5 (**a**, **b**, **d**, **e**) and E17.5 (**c**, **f**). Sortilin localizes prominently to the epithelium and glands of the nasal cavity (**a**, **c**) and is also found in the tracheal epithelium (**b**). Furthermore, sortilin is highly expressed in the apical part of the epithelium of the branching bronchial network at all three time points investigated. The expression is confined primarily to distal branches and is diminished in proximal more mature bronchi (**d**, **e**). Green channel is sortilin, red channel is calretinin (**a**) or TTF-1 and blue channel is Hoechst nuclear stain. Scale bars = 50 μm. Br, bronchus; Ca, cartilage; Db, distal branch; Gl, epithelial gland; Na, nasal cavity; Ol, olfactory epithelium; Pl pleuric cavity; Tr, tracheal epithelium

At all three ages E13.5, E15.5 and E17.5, moderately strong sortilin immunoreactivity (IR) was found in the epithelium of the nasal cavity. The expression was localized to the apical part of the epithelial cells and showed a granular pattern. At E15.5, cells in the epithelium marked by the calcium-binding protein calretinin, a marker for maturing olfactory cells [28], showed sortilin IR as well (Fig. 1a, b). In the respiratory part of the pharynx as well as the larynx and trachea, sortilin IR was again found in the apical part of the epithelial cells, albeit at a weaker level. This pattern was repeated through E13.5-E17.5. Lastly, strong expression of sortilin was found in epithelial glands of the nasal cavity at E17.5 (Fig. 1c).

##### Lung/Lower airways

Sortilin was found highly expressed in the developing lung, and showed distinct spatial distribution throughout all three age points. At E13.5, in the pseudo glandular lung stage, sortilin reactivity was found to be pronounced in the epithelium of the entire developing bronchial system as marked by Thyroid Transcription Factor 1 (TTF-1), a marker for developing bronchial cells [29]. Expression had a granular pattern and was most prominent in the apical part of the epithelial cells (Fig. 1d, *d*'). The mesenchyme showed very sparse reactivity. At E15.5, in the early canalicular phase, the pattern was similar, albeit with a lower expression of sortilin in more proximal bronchi. At E17.5 in the beginning of the saccular phase the pattern of sortilin had changed somewhat. In the more distal branches of the tubular system sortilin was highly expressed in the apical vesicular pattern previously described. However, in the parts of the airways resembling developing alveoli, sortilin reactivity was very sparse (Fig. 1e, f). Furthermore, the mesenchyme/stroma began to show distinct sortilin IR. Along the borders of the prospective alveoli and bronchioli, solitary cells with a round soma approximately 10–12 μm in diameter and strong granular expression of sortilin were found. These cells resembled macrophages.

**Fig. 2:**
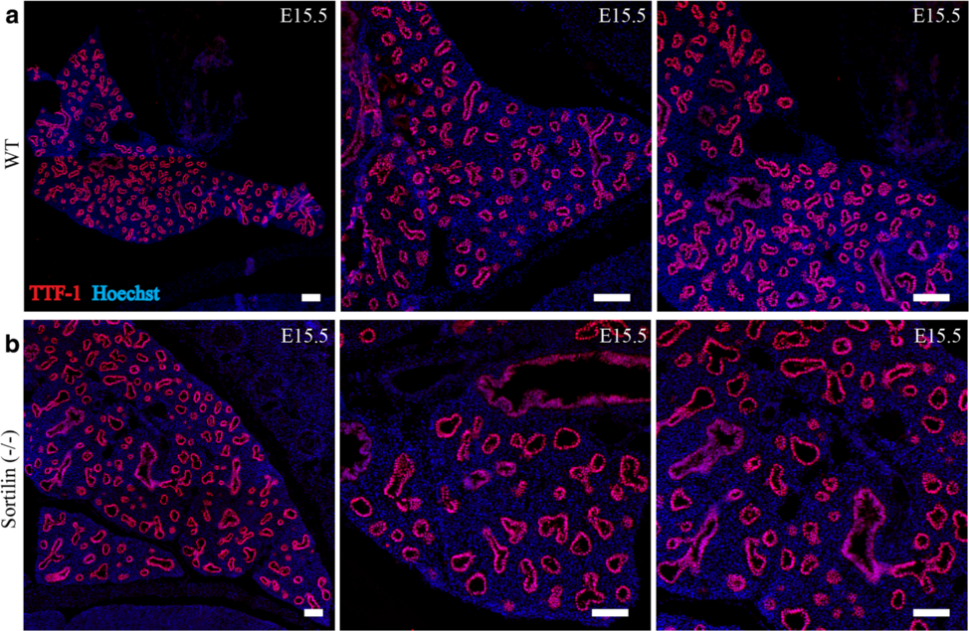
Sortilin (−/−) embryos might have fewer and larger lung branches. Preliminary data. Embryos were taken at E15.5 from WT and Sortilin embryos, and cut at 10 μm thick slices on a cryotome and stained for TTF-1 (*Red channel*) and Hoechst (*Blue channel*). **a** shows images from WT lung at E15.5 and **b** shows lung from a Sortilin(−/−) embryo. The bronchial branches appear larger and fewer in the knockout compared to the WT, resembling a more immature lung (see pictures from E13.5 in Fig. 1 for comparison). Scale bars = 100 μm

A final note on lungs, preliminary examination of two Sortilin(−/−) embryos give the impression that at E15.5 the lung epithelium have fewer branches per lung area and have a larger lumen compared to (two) WT mice. A comparison is shown in Fig. 2 but this must be checked in many more mice before a statistically relevant conclusion can be drawn.

In short summary, sortilin IR in the developing respiratory system was largely confined to the apical parts of epithelia, most pronounced in the roof of the nasal cavity and in the presumptive more immature stages/parts of the developing lower airways.

### Digestive tract

#### Mouth and esophagus

At E13.5 the epithelium of the mouth and tongue both showed sortilin IR at a weak level, stronger in the latter (Fig. 7a, b). By E15.5 this expression had diminished, apart from a few epithelial cells dispersed in the posterior palate, and by E17.5, no sortilin-IR could be detected. In the esophagus, weak sortilin IR in the epithelium was found consistently at all the three age points.

#### Submandibular gland

**Fig. 3:**
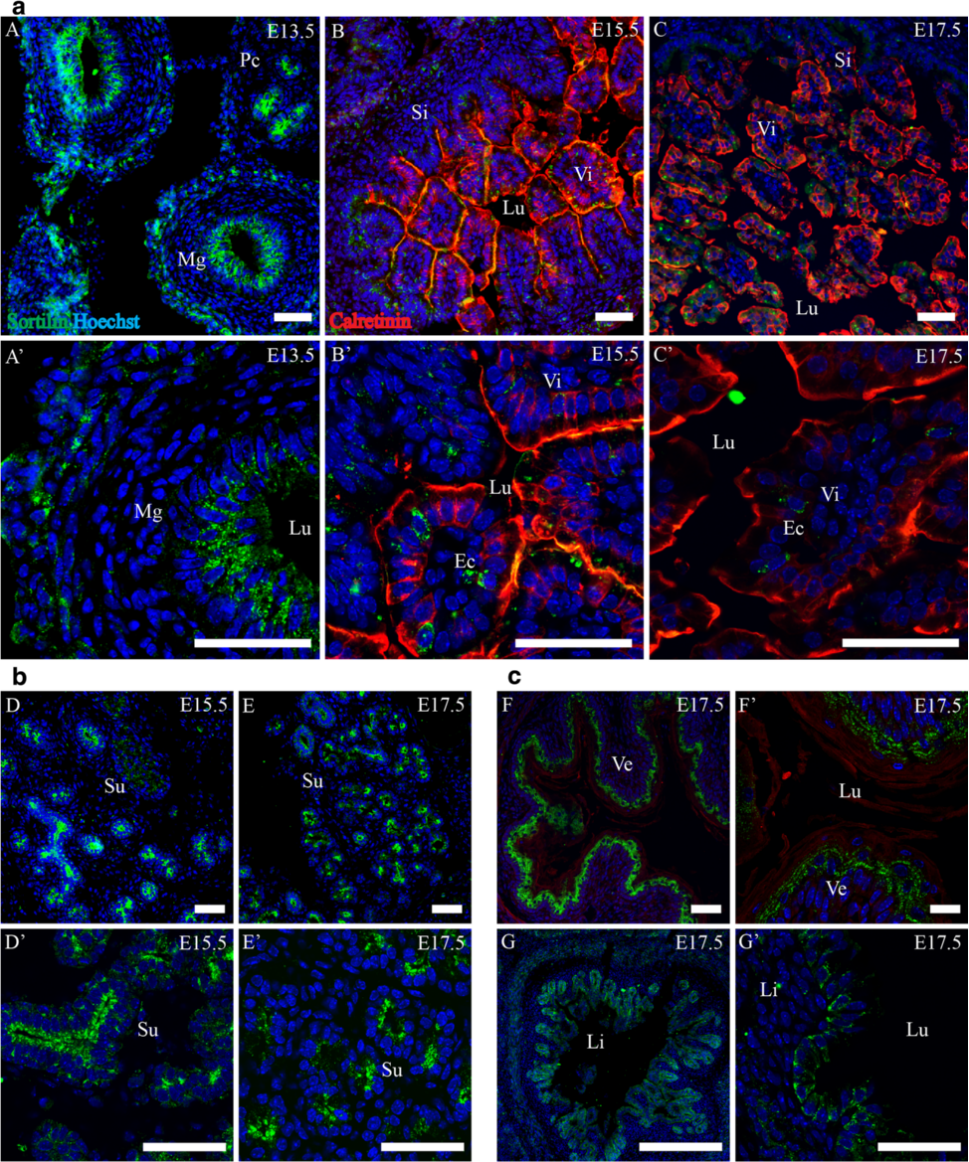
Expression of Sortilin in the developing alimental canal and submandibular gland. Immunohistofluorescence was performed on sagittal sections (10 μm) of embryos on embryonic day E13.5, E15.5 and E17.5. **a**. Sortilin is expressed in the epithelium of the midgut at E13.5 (*a*, *a*’) but is not observed in the small intestine from E15.5 and onwards, apart from a small subset of epithelial cells (*b*-*c*’). **b**. Sortilin is highly expressed in the epithelium of the developing submandibular gland and is primarily apically located (*d*-*e*’). **c**. Sortilin is moderately expressed in the epithelium of the developing ventricle (*f*, *f*’) and the large intestine at E17.5 (*g*, *g*’). Green channel is sortilin, red channel is calretinin and blue channel is Hoechst nuclear stain. Scale bars = 50 μm. Ec, presumptive enteroendocrine cell; Li, large intestine; Lu, lumen; Mg, midgut; Pc, pancreas; Si, small intestine; Su, submandibular gland branches; Ve, ventricle; Vi, intestinal villus

Sortilin showed a distinct expression pattern in the submandibular gland. Expression was confined to the apical part of the epithelial cells of acini as well as ducts in a granular manner at E13.5. This persisted throughout the course of E15.5-E17.5, as the gland grew larger and more complex (Fig. 3b).

#### Gut

At E13.5 sortilin IR was found in midgut, most pronounced in the epithelium, as well as in a circular band with a thickness of 2–3 cells in the periphery of the gut wall. In the epithelial cells, sortilin IR was located apically in a granular pattern (v 3a). At E15.5 sortilin IR of the gut had decreased. In the developing small intestine, expression of sortilin was present weakly in the epithelium, and strongly in a subset of cells herein. In these cells sortilin was often located basally to the nucleus in resemblance of enteroendocrine cells, and sometimes in a small cellular process extending along the basal epithelial border (Fig. 3a, *b*' ,*c*'). In the large intestine, sortilin IR was noted in the epithelium at a moderate level (Fig. 3c). The same pattern of expression was apparent at E17.5 with the addition of expression in the ventricular epithelium.

#### Liver

**Fig. 4:**
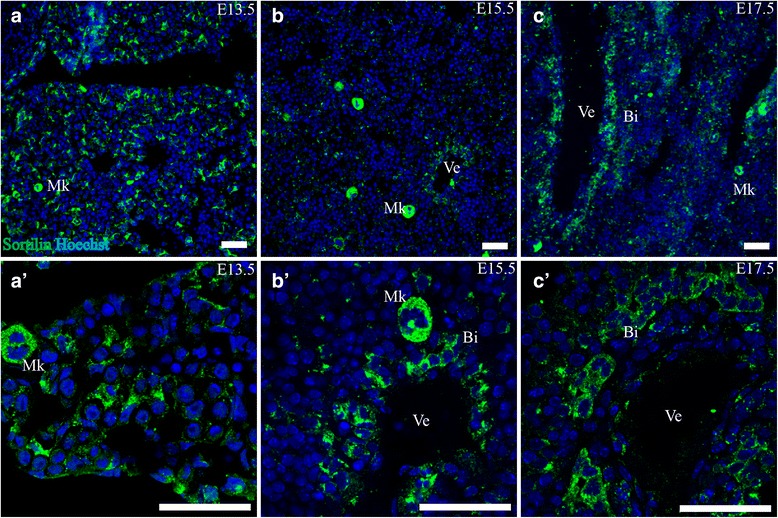
Sortilin expression in liver. Immunohistofluorescence was performed on sagittal sections (10 μm) of embryos on embryonic day E13.5 (**a**,*a*’), E15.5 (**b**, *b*’) and E17.5 (**c**, *c*’). Sortilin-IR was found in megakaryocytes of all three age points, as well as in structures morphologically identified as immature bile ducts in the vicinity of veins. Green channel is sortilin and blue channel is Hoechst nuclear stain. Scale bars = 50 μm. Bi, developing bile duct; Mk, megakaryocyte; Ve, presumptive vein

Throughout all three stages investigated, similarities in sortilin IR were found in the liver. Large cells with intense granular expression of sortilin were found across the organ. These cells were up to 25 μm in diameter with a large lobed nucleus resembling megakaryocytes. Additionally, a strong granular expression of sortilin was present in cells harboring a nucleus with several lobes, resembling neutrophil granulocytes, as well as in cells with a bended nucleus resembling eosinophils or monocytes. Specifically for E13.5, sortilin IR was found in cells with an elongated nucleus and cytoplasm that was often surrounding a single cell or a few identical looking blood cells of various lineages. At E15.5 and E17.5, clusters of sortilin IR cells in the near vicinity of large vessel-like structures were present. These clusters often had a bi-layered structure surrounding a small lumen, fitting the morphology of developing intrahepatic bile ducts [30]. Liver findings are shown in Fig. 4.

#### Pancreas

**Fig. 5:**
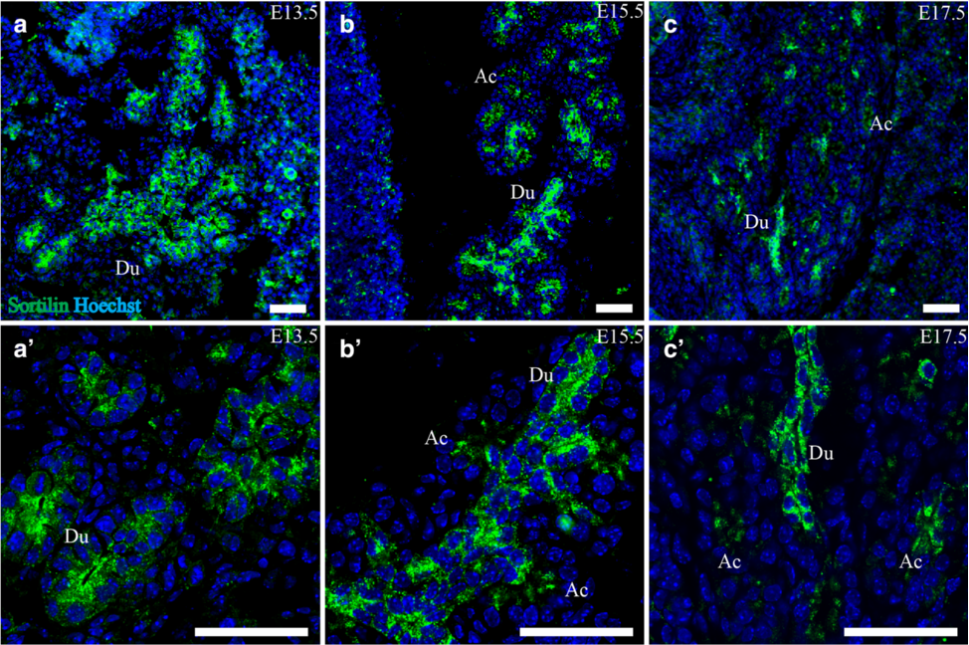
Sortilin is expressed in the epithelium of the developing pancreas. Immunohistofluorescence was performed on sagittal sections (10 μm) of embryos on embryonic day E13.5 (**a**, *a*’), E15.5 (**b**, *b*’) and E17.5 (**c**, *c*’). Sortilin is highly expressed in the ductal epithelium, and to a lesser extent in the apical part of developing acini. Green channel is sortilin and blue channel is Hoechst nuclear stain. Scale bars = 50 μm. Ac, acini; Du, ductal system

In the pancreas, sortilin IR was localized to the parenchyma of the exocrine part. At E13.5, the epithelium of the ductal system showed high sortilin IR, primarily in the apical part and of granular appearance (Fig. 5). This was also true for E15.5 and E17.5, where the exocrine acini also showed granular sortilin IR in the apical parts of the acinar cells, although not as marked as the ductal cells.

In short summary, sortilin IR in the digestive system was found at the highest levels in the epithelium of the submandibular gland acini and ducts, and the secretory ducts of pancreas, but was also highly expressed in the liver in granulocytes, megakaryocytes and developing bile ducts. Lastly sortilin IR was found in cells resembling enteroendocrine cells of the gut as well as in epithelia of the ventricle and large intestine.

### Urinary system

#### Kidney

**Fig. 6:**
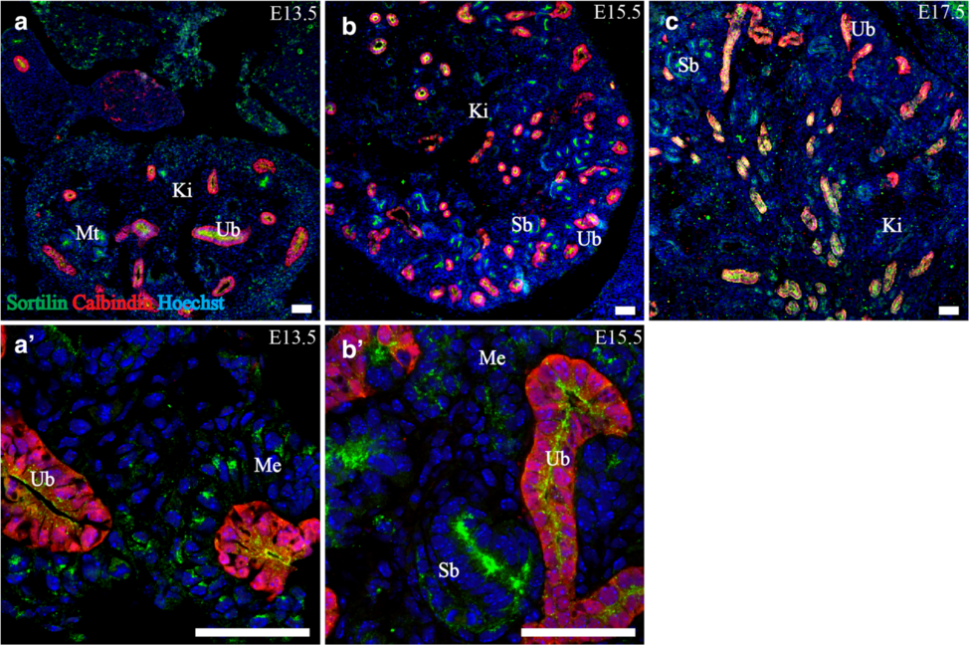
Sortilin is expressed in the tubular system of the developing metanephros. Immunohistofluorescence was performed on sagittal sections (10 μm) of embryos on embryonic day E13.5 (**a**, *a*’), E15.5 (**b**, *b*’) and E17.5 (**c**). Sortilin shows marked apical expression in ureteric branches and a portion of the metanephric tubular system. This expression becomes more prominent from E13.5-E15.5 and remains stable at E17.5. Green channel is sortilin, red channel is Calbindin-D28K and blue channel is Hoechst nuclear stain. Scale bars = 50 μm. Ki, kidney; Me, metanephric mesenchyme; Sb, S-shaped body Ub, ureteric branch

Sortilin was found expressed in the tubular system inside the metanephros at all three age points. At E13.5 sortilin IR was pronounced in cells expressing Calbindin-d28k, a marker for ureteric branches [31], and developing nephrons (Fig. 6). Furthermore, metanephric tissue surrounding the ureteric buds showed expression of sortilin. At E15.5 sortilin was differentially expressed in the epithelial tubular system, with sparse reactivity in the mesenchyme. Most ureteric branches, now developing into collecting ducts, showed a similar expression pattern to that of E13.5. Some nephric tubules showed a strong granular pattern of sortilin IR, and these had a high cuboidal-cylindrical epithelium with sortilin predominantly located in the apical part. Other tubules with a lower, flattened cuboidal epithelium showed weaker sortilin IR. In developing renal corpuscles, weak-moderate sortilin IR was found along the parietal layer of Bowman’s capsule (not shown), as well as in a few cells dispersed in the glomerulus. At E17.5 the pattern was similar to E15.5.

### Heart and vascular system

#### Heart

**Fig. 7:**
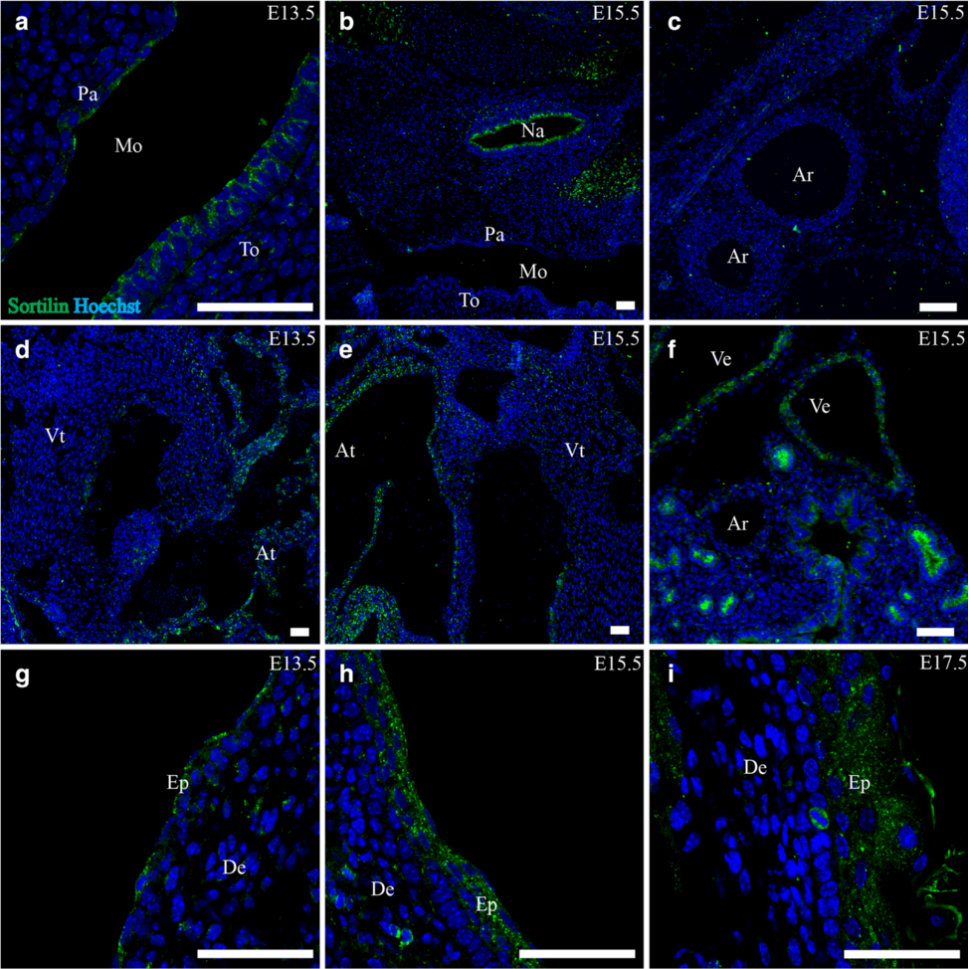
Sortilin expression in mouth, heart, vascular system and skin. Immunohistofluorescence was performed on sagittal sections (10 μm) of embryos on embryonic day E13.5, E15.5 and E17.5. Sortilin is found in the epithelium of the tongue and palate at E13.5 but is gone by E15.5 (**a**,**b**). In the cardiovascular system, sortilin is found in the atria and large veins (**d**-**f**) but not in the arteries (**c**), Finally, sortilin is found moderately expressed in the epidermal layers of the skin most prominently at E15.5 (**g**-**i**), Green channel is sortilin, and blue channel is Hoechst nuclear stain. Scale bars = 50 μm. Ar, arteries; At, atria of heart; De, dermis; Ep, epidermis; Mo, mouth cavity; Na, nasal cavity; Pa, epithelium of palate; To, tongue; Ve, veins, Vt, ventricle of heart

Expression of sortilin in the heart showed a similar pattern throughout E13.5, E15.5 and E17.5. Very sparse reactivity was found in the ventricle, but contrary to this, a moderately strong expression was apparent in the atria (Fig. 7d, e). This spanned the wall as well as the valves.

#### Large blood vessels

In the vascular system, the arteries showed no expression of sortilin while the veins showed moderate to strong expression at all three age points (Fig. 7c, f).

### Musculoskeletal system

#### Cartilage/Bone

**Fig. 8:**
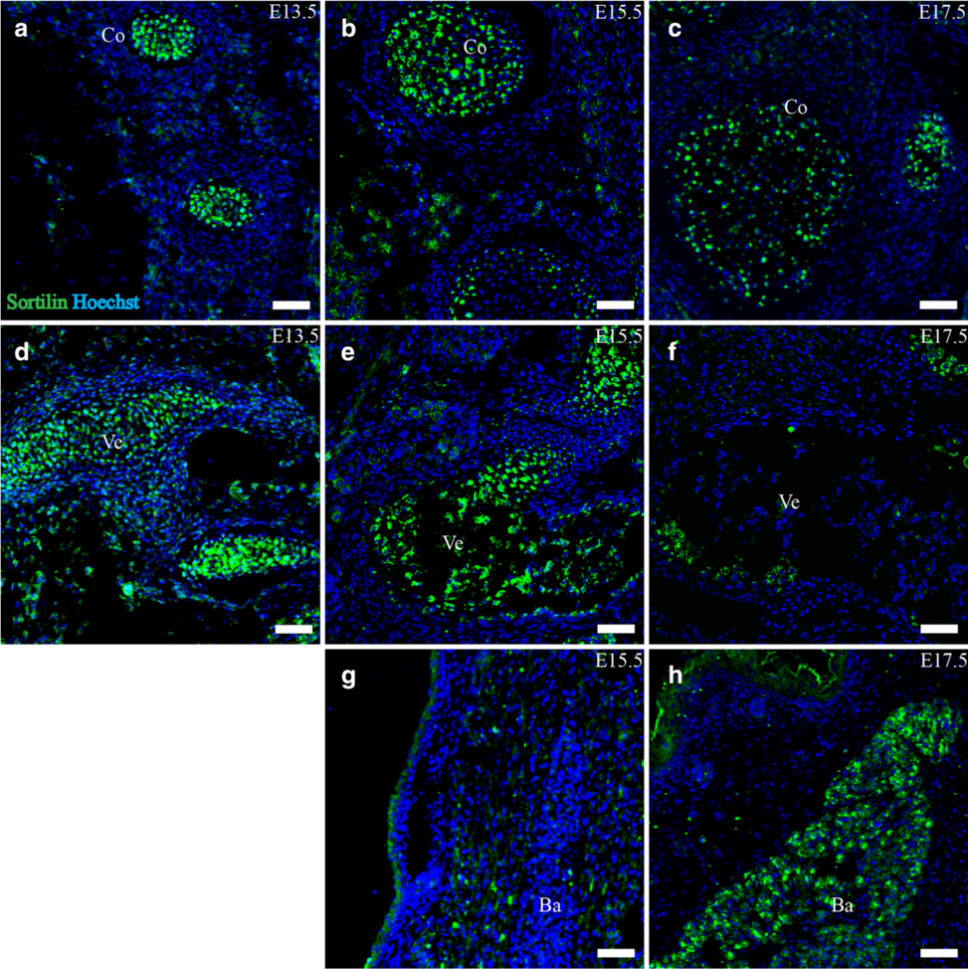
Sortilin is expressed in costae, vertebrae and brown adipose tissue. Immunohistofluorescence was performed on sagittal sections (10 μm) of embryos on embryonic day E13.5, E15.5 and E17.5. Sortilin show marked central expression in developing costae (**a**-**c**), as well as in the chondral primordium and later ossifying vertebrae (**d**-**f**). Sortilin expression is absent from brown adipose tissue until E17.5 where it is prominent (**g**,**h**). Green channel is sortilin and blue channel is Hoechst nuclear stain. Scale bars = 50 μm. Ba, brown adipose tissue; Co, costae; Ve, vertebrae

Sortilin IR was generally detected at high levels in the entire skeletal primordium as well as cartilage. In structures undergoing intramembranous ossification e.g. skull, sortilin was expressed in the entire structure. In structures undergoing endochondral ossification e.g. costae, sortilin expression was generally confined to cells in the center of the structure, an area with more space between nuclei compared to the surrounding concentric cell layers (Fig. 8a-c).

At E15.5, sortilin IR was pronounced in the cartilage anlagen of the long bones, facial bones and vertebrae. Furthermore sortilin was strongly expressed in costae, cartilage of the larynx, trachea and the skull. Expression was confined to the central cells as described for E13.5. Furthermore, areas undergoing ossification in the spine showed strong sortilin expression in a centered pattern (Fig. 8d, e).

At E17.5, cartilage of the trachea and larynx as well as costae showed similar sortilin reactivity to earlier age points. In the ossifying spine, the pattern of expression had changed somewhat. Sortilin IR cells were no longer found in the center, but rather in the posterodorsal and anteroventral parts of the vertebrae, confined to a cluster of cells: some cells with a diameter of ~10 μm, and other large cells at ~15–20 μm in diameter (Fig. 8f). These cells are possibly osteoblasts and osteoclasts, respectively, where both showed a strong granular pattern of sortilin expression dispersed throughout the cytoplasm.

#### Skin

The skin showed little sortilin IR at E13.5, but this increased to moderate levels at E15.5 and E17.5. Expression was located in the apical part of the epidermis (Fig. 7g-i).

#### Adipose tissue

At E13.5 and E15.5, adipose tissue showed no expression of sortilin. By E17.5, the brown (multilobular) fat deposits in the neck and back region showed strong sortilin IR, with a peak along the edges of deposits (Fig. 8g, h)

In summary, sortilin expression was found pronounced in skeletal structures undergoing both endochondral and intramembranous ossification, as well as in cartilage of e.g. larynx. Furthermore brown fat deposits showed strong sortilin IR by E17.5.

### Endocrine system

#### Thyroid gland

**Fig. 9:**
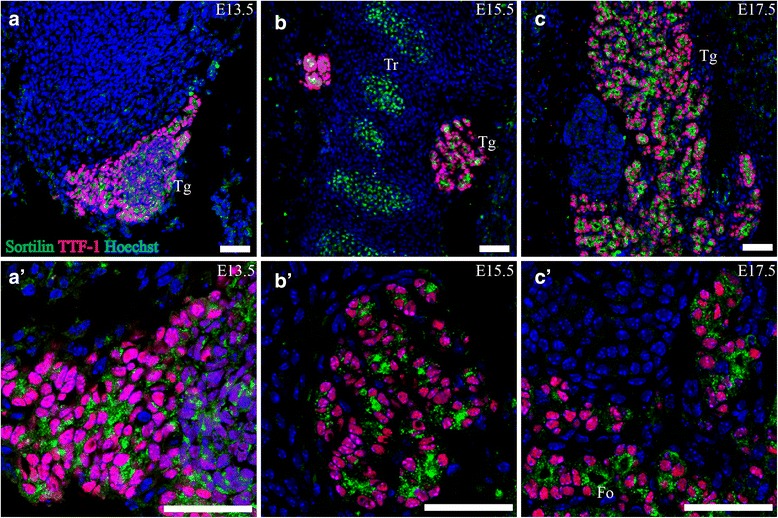
Sortilin expression in the developing thyroid gland. Immunohistofluorescence was performed on sagittal sections (10 μm) of embryos on embryonic day E13.5 (**a**, *a*’), E15.5 (b, *b*’) and E17.5 (**c**, *c*’). Sortilin is highly expressed in TTF-1 IR cells in the developing thyroid gland on all age points investigated. On E17.5 the expression was localized to the apical part of TTF-1 IR cells forming follicles (**c**, *c*’). Green channel is sortilin, red channel is TTF-1 and blue channel is Hoechst nuclear stain. Scale bars = 50 μm. Fo, emerging follicles; Tg, thyroid gland; Tr, tracheal cartilage

At E13.5, the thyroid gland showed strong sortilin IR in cells expressing TTF-1 (Fig. 9). By E15.5, folliculogenesis has commenced, and TTF-1 IR cells have begun to arrange in small groups with sortilin IR in the part of the cells facing the center. At E17.5, small follicles are clearly visible, with strong granular sortilin expression in the apical cytoplasm of TTF-1 IR cells facing the emerging lumen.

#### Adrenal glands

In the developing adrenal glands, expression of sortilin was noted at E13.5 in cells staining positive for tyrosine hydroxylase (TH). Furthermore, cells surrounding the TH positive cells in the adrenal glands also showed sortilin IR. This expression diminished to non-detectable levels at E15.5 and E17.5.

### SorCS2

#### Respiratory tract

##### Lung

**Fig. 10:**
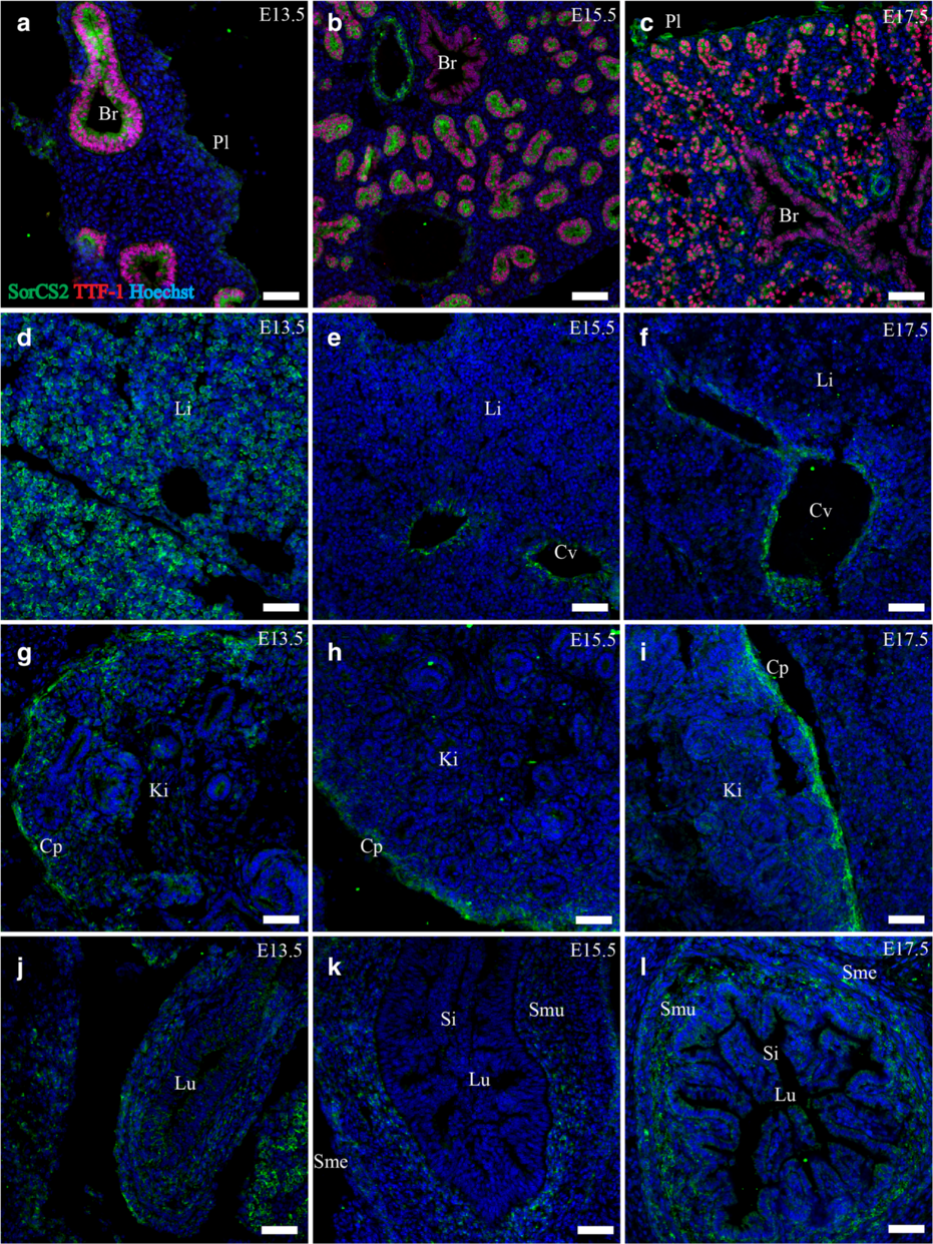
SorCS2 expression in embryonic internal organs. Immunohistofluorescence was performed on sagittal sections (10 μm) of embryos on embryonic day E13.5, E15.5 and E17.5. SorCS2 is found prominently in the epithelium of the developing lung, and also the visceral pleural layer (**a**-**c**) It is highly expressed in the liver at E13.5 (**d**), but diminishes at later time points and instead localizes to central veins (**e**,**f**). SorCS2 is also expressed in the cap mesenchyme of the kidney (**g**-**i**), and in the connective tissue of the gut (**j**-**l**). Green channel is SorCS2, red channel is TTF-1 and blue channel is Hoechst nuclear stain. Scale bars = 50 μm. Br, bronchus; Cp, Cap mesenchyme; Cv, central vein; Ki, kidney; Li, liver; Lu, lumen; Pl pleuric cavity; Si, small Intestine; SMe, submesothelial connective tissue; SMu, submucosal connective tissue

SorCS2 was found expressed at high levels in the apical parts of developing bronchi analogous to the expression of sortilin, throughout E13.5, E15.5 and E17.5, with the exception that SorCS2 showed a less pronounced granular pattern. The visceral blade of the pleura also stained clearly for SorCS2 (Fig. 10a-c).

#### Digestive tract

##### Gut

Moderate expression of SorCS2 was found in the connective tissue of the lamina propria and submucosal layer of the developing gut at E15.5 and E17.5 as well as the submesothelial connective tissue (Fig. 10j-l).

##### Liver

The liver showed strong expression of SorCS2 at E13.5. The cells expressing SorCS2 were spherical with a round nucleus and sparse cytoplasm around 10 μm in diameter and SorCS2 was located perinuclear in a granular pattern. The cells were often located together in small groups interspersed by areas of cells without expression of SorCS2 (Fig. 10d). By E15.5 and E17.5 this pattern of expression had diminished to very low levels. At E15.5 and E17.5 expression of SorCS2 is noted in vessel-like structures corresponding to central veins (Fig. 10e, f).

#### Urinary system

##### Kidney

Throughout all three time points there was a moderate expression of SorCS2 in the periphery of the developing kidney, corresponding to the cap mesenchyme/cortical interstitium. The level of this expression remained fairly constant throughout the three time points investigated (Fig. 10g-i).

#### Heart and vascular system

##### Heart

**Fig. 11:**
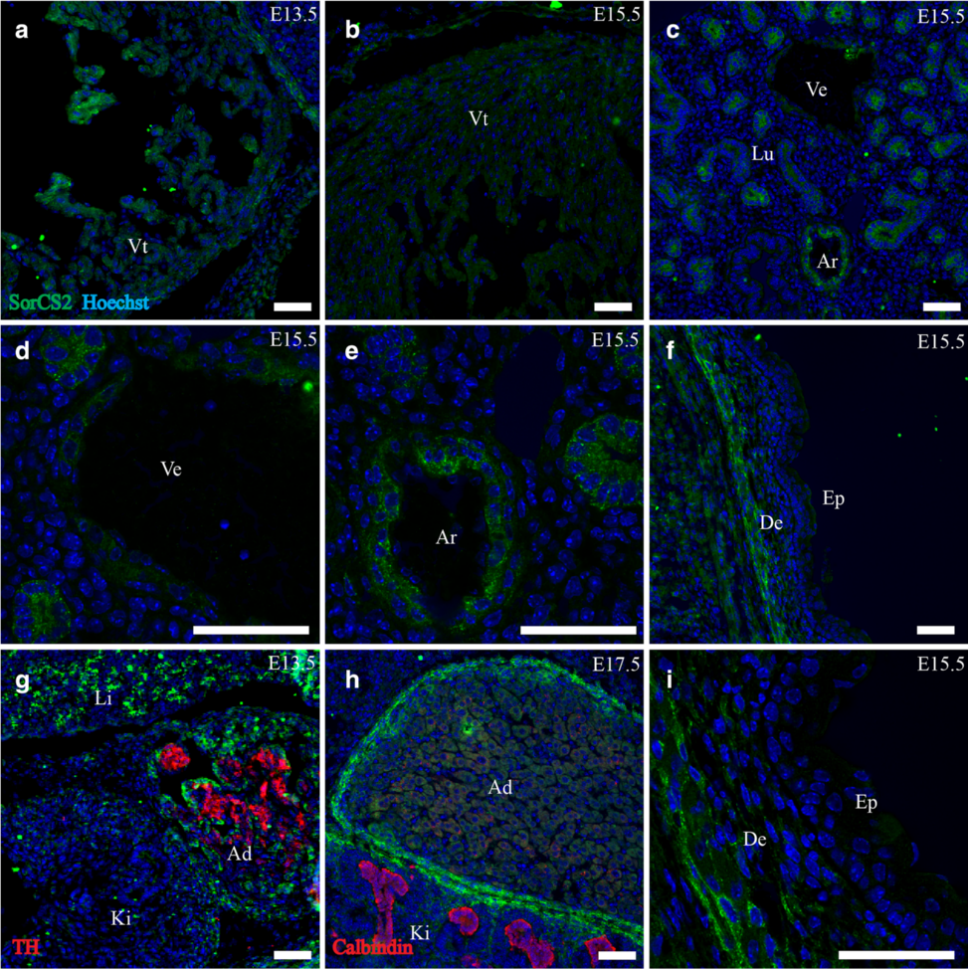
SorCS2 expression in cardiovascular system, skin and adrenal gland. Immunohistofluorescence was performed on sagittal sections (10 μm) of embryos on embryonic day E13.5, E15.5 and E17.5. SorCS2 is weakly expressed in the developing ventricle in a subset of cells (**a**, **b**). Medium-large arteries show prominent SorCS2 IR while veins show weak IR (**c**-**e**). In the skin SorCS2 is found localized to the deep dermal layers (**f**, **i**). Lastly, the adrenal gland shows pronounced expression of SorCS2 in a large portion of the cells (**g**) but by E17.5 this localization has changed to the periphery of the gland, analogous to the kidney (**h**). Green channel is SorCS2, red channel is TH (**g**) or calbindin (**h**) and blue channel is Hoechst nuclear stain. Scale bars = 50 μm. Ad, adrenal gland; Ar, arteries; De, dermis; Ep, epidermis; Ki, kidney; Lu, lung; Ve, vein; Vt, ventricle of heart

The ventricles and atria of the developing heart both showed weak levels of expression of SorCS2 at E13.5. This expression was diminished at E15.5 and was non-detectable at E17.5 (Fig. 11a, b).

##### Vessels

Prospective small arterioles and medium-large arteries showed prominent expression of SorCS2 in both the systemic and pulmonic circulation. SorCS2-IR localized to concentric cell layers in the vessel wall disparate from the endothelium (Fig. 11c–e). Larger veins also showed faint expression of SorCS2 in the same pattern.

#### Musculoskeletal system

##### Cartilage/Bone

**Fig. 12:**
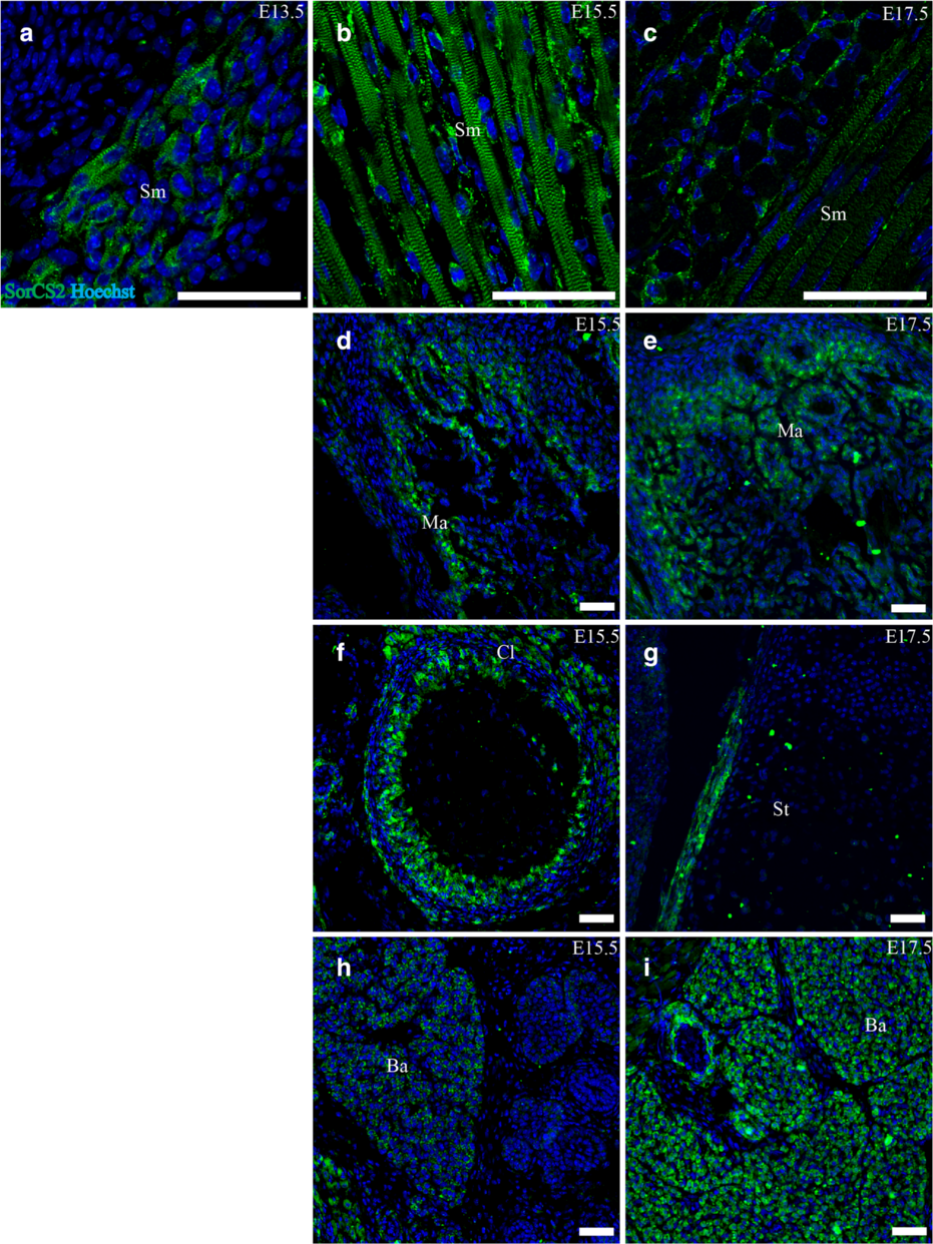
SorCS2 is expressed in striated muscle, costae, mandible and adipose tissue. Immunohistofluorescence was performed on sagittal sections (10 μm) of embryos on embryonic day E13.5, E15.5 and E17.5. **a**-**c**. SorCS2 is expressed in striated muscle at all age points, localizing both in satellite cells and in striated bands with a diminishing trend at E17.5. **d**-**g**. SorCS2 shows marked concentric expression in developing costae, as well as in the ossifying mandible. **h**,**i**. SorCS2 is also expressed in brown adipose tissue. Green channel is SorCS2 and blue channel is Hoechst nuclear stain. Scale bars = 50 μm. Ba, brown adipose tissue Cl, clavicle; Ma, mandible; Sm, striated muscle; St, sternum

SorCS2 was prominently expressed in ossifying bone such as the mandible and vertebrae at E15.5 and E17.5. The expression localized mostly to the outer 1/3 of the ossifying area. Furthermore, SorCS2 was prominently expressed in a concentric layer of mesenchyme surrounding the costae, sternum, clavicle and skull (Fig. 12d, e).

##### Striated muscle

Throughout E13.5, E15.5 and E17.5 SorCS2 was found expressed at high levels ubiquitously in striated muscle. The expression located mainly in satellite cells closely associated with muscle cells but also directly to one of the repeating bands of sarcomeres in muscle cells. The sarcomere part diminished at E17.5 (Fig. 12a-c).

##### Skin

The connective tissue of the dermal layers of the skin had pronounced expression of SorCS2 at E15.5 and E17.5 (Fig. 11f, i).

##### Adipose tissue

SorCS2 was found expressed abundantly in the brown multilobular adipose tissue of the neck at all three age points (Fig. 12h, i).

#### Endocrine system

##### Adrenal glands

Expression of SorCS2 was prominent in the developing adrenal gland at E13.5, confined to cells in close proximity to TH+ chromaffin progenitor cells. This corresponds to the steroidogenic cell population. Furthermore expression of SorCS2 was present in the peripheral mesenchyme. By E15.5 the expression in the steroidogenic cell population had diminished to low levels and was undetectable at E17.5 (Fig. 11g, h).

## Discussion

In the CNS, sortilin and SorCS2 show a complementary expression with regards to cell type and subcellular distribution [2]. This disparate expression pattern might indicate overlapping roles for sortilin and SorCS2 in the CNS, in addition to their unique functions. Indeed, the ability of both proteins to mediate apoptotic signals in the CNS could mean that the some of the differences in the amino-acid sequence of sortilin and SorCS2 help suit them to mediate similar effects in dissimilar cell types. As such it was interesting to see if these largely complementary expression patterns were also found in non-neuronal tissues of the developing embryo. This indeed seemed to be the case, as sortilin was mainly found in epithelial tissues, whereas SorCS2 predominated in mesodermally derived connective tissues.

### Sortilin in the developing embryo

Sortilin has been shown to bind multiple ligands, and to have physiological functions beyond the nervous system. Possible roles for sortilin in the development of the embryo have not been examined. We show sortilin to be abundantly expressed in the developing embryo in a surprisingly large number of organs and tissues. The most consistent finding is that sortilin is located to the epithelial tissues, and more specifically in the apical part of these. Many of these epithelial structures undergo significant branching in the period examined, notably lung, kidney, pancreas and the submandibular gland. The branching process is conceivably highly regulated and involves a variety of intrinsic and extrinsic mechanisms that work together to ensure the proper architecture of the given organ [32]. Among these mechanisms are several signaling molecules of well-known growth factor systems including members of the fibroblast growth factor family and bone morphogenetic proteins. During epithelial differentiation, there is a substantial amount of receptor regulation, where epithelia lose and acquire sensitivity to different extrinsic signals as they differentiate into more mature cell types. Sortilin is a multi-ligand receptor shown to function at the level of the trans Golgi network (TGN) in sorting a variety of molecules between different cellular compartments [3]. Sortilin could participate in the trafficking and regulation of various receptors or effector molecules involved in the branching process of these organs. The preliminary data from the lung of a sortilin(−/−) embryo could indicate a reduced level of branching, with fewer and larger bronchial branches compared to WT, however this needs to be confirmed with a more thorough investigation. Interestingly, sortilin has been shown to interact with transforming growth factor beta (TGFβ) and increase its degradation [33]. A study using a constitutively active form of TGFβ-1 in fetal mice showed arrest of lung development in the pseudo glandular stage, and lack of sortilin could hypothetically result in increased TGFβ signaling leading to delayed lung maturation [34]. The temporal shift in sortilin expression noted in the lung is in line with a hypothesis of sortilin playing a role in the branching process of this organ.

The developing thyroid gland showed strong sortilin expression through all three age points. Interestingly sortilin has been shown to bind thyroglobulin, the precursor protein of thyroid hormones T3 and T4 [35]. Thyroglobulin is secreted from the apical side of thyroid epithelia, the tyrosine residues are modified after which thyroglobulin is taken up again by endocytosis and proteolysed in lysosomes. Despite this, later investigations have showed that the levels of TSH and thyroglobulin as well as the morphology of the thyroid gland are normal in sortilin(−/−) mice. The only difference found was reduced T4 levels in male sortilin(−/−) mice [36]. Whether this is due to redundancy of sortilin in thyroid gland function, or a question of experimental circumstances remain to be discovered.

Another tissue with prominent sortilin IR is cartilage and bone. Both types of tissues produce and secrete large amounts of extracellular proteins regulated by extracellular signals, and sortilin could be involved in the substantial intracellular trafficking needed to effectuate this [37, 38]. Furthermore, sortilin is expressed in several other tissues ranging from blood cells to brown adipose tissue.

Earlier studies have examined the expression of sortilin and SorCS2. Petersen et al. investigated the expression of sortilin in adult human tissues by northern blotting [39]. They found expression of two different mRNA transcripts of 8 and 3.5 kb. The 8 kb transcript was found in high amounts in heart and skeletal muscle, thyroid gland, placenta and testis and in minor amounts in kidney, colon, liver and lymphoid organs. Hermans-Borgmeyer et al. have investigated the expression of sortilin mRNA in mouse embryological development with focus on the central nervous system. In the peripheral organs they found the sortilin mRNA transcripts most abundantly expressed in lung and kidney, but also in heart and skeletal muscle [40]. Sarret et al. investigated the distribution of Sortilin mRNA and protein in rat central nervous system. They found that sortilin predominantly localized in cell somata as well as in the proximal dendrites of neurons [41]. Generally, this is well in line with our findings with the exception of Petersen et al. who investigated adult human tissue. While the expression of sortilin in adult human vs fetal mouse tissue can be genuinely different there may be other explanations as well. The localization of mRNA transcripts and the protein sortilin are not necessarily the same, which could also be the reason why Hermans-Borgmeyer et al. find expression of sortilin in skeletal muscle in contrast to us. Furthermore, Petersen et al. do not state whether they had other human tissues available so it is possible that they would find expression also in lung, pancreas, salivary glands and so forth.

### SorCS2

Contrary to sortilin, SorCS2 was found expressed prominently in mesodermally derived structures such as adipose tissue, striated muscle tissue and developing bone. This could indicate a function for SorCS2 in these tissues. A genome wide association study found that a single nucleotide polymorphism in the locus of the SorCS2 gene was significantly associated with circulating levels of insulin-like growth factor binding protein 3 (IGFBP-3) in a human population [42]. IGFBP-3 binds and regulates the bioavailability of IGF-1 in plasma, and overexpression in a mouse model showed growth retardation [43]. IGF-1 is a primary effector of growth hormone, and stimulates growth of most tissues including muscle, adipose tissue and bone [44]. Thus, it is interesting to investigate whether SorCS2(−/−) show signs of reduced growth. Furthermore, SorCS2 was found expressed in several connective tissues, such as the dermis, submucosal and submesothelial layers of the gut. Finally SorCS2 was also found in the epithelium of the bronchial system, which differs somewhat from the general localization.

Rezgaoui et al. have investigated the expression of SorCS2 in mouse embryonic tissue by in situ hybridization in CD-1 mice. They found SorCS2 transcripts localized to facial mesenchyme, skeletal and smooth muscle, cartilaginous tissue and lung epithelial tissue. Furthermore, they also found weak levels of expression in heart muscle and olfactory epithelium. This corresponds well with our findings, apart from the SorCS2 protein we see in adipose tissue. This could be due to methodological differences as mentioned above, or to differences in gene expression between CD-1 and C57J/bl6bom mice.

The complementary expression pattern of sortilin and SorCS2 in some body parts raises the question whether this also translates to complementary functions in these tissues. This seems to be the case for dorsal root ganglion neurons and Schwann cells, which require sortilin and SorCS2 respectively, for p75^NTR^ mediated apoptosis [2]. SorCS2 differs from sortilin in that it does not have complete cytoplasmic tail sorting motifs needed to convey transport between the TGN and lysosomes [45]. Thus it is possible that SorCS2 does not share functional similarities in this aspect, but rather in functions on the cell surface. It should be noted that the complementary pattern described is not complete, as there are several organs that contain sortilin but not SorCS2 e.g. pancreas, submandibular gland, and organs that contain both proteins in the same cells (lung).

## Conclusions

The developing mouse embryo shows an extensive and distinct expression of sortilin and SorCS2 in a large variety of tissues, with several dissimilar physiological roles possible. It is our hope that this structural characterization will encourage new studies of the functions of sortilin and SorCS2 in organ development.

### Availability of data

Additional images are available on request. Information on the performance of antibodies for IHC was deposited in the open access database pAbmAbs (http://www.pabmabs.com) under Simon Bøggild or Simon Mølgaard, Aarhus University.
